# Supplementary material for: Heavy fermions vs doped Mott physics in heterogeneous Ta-dichalcogenide bilayers
Source: Nat Commun. 2024 Feb 14;15:1357. doi: 10.1038/s41467-024-45392-y (PMC10866876; doi:10.1038/s41467-024-45392-y)
Supplement: Supplementary file 1 — Supplementary Information [file 41467_2024_45392_MOESM1_ESM.pdf]

Supplementary Information for Heavy Fermions vs doped Mott physics in heterogeneous Ta-dichalcogenide bilayers

SUPPLEMENTARY NOTE 1. 1H CHARGE DENSITY WAVE

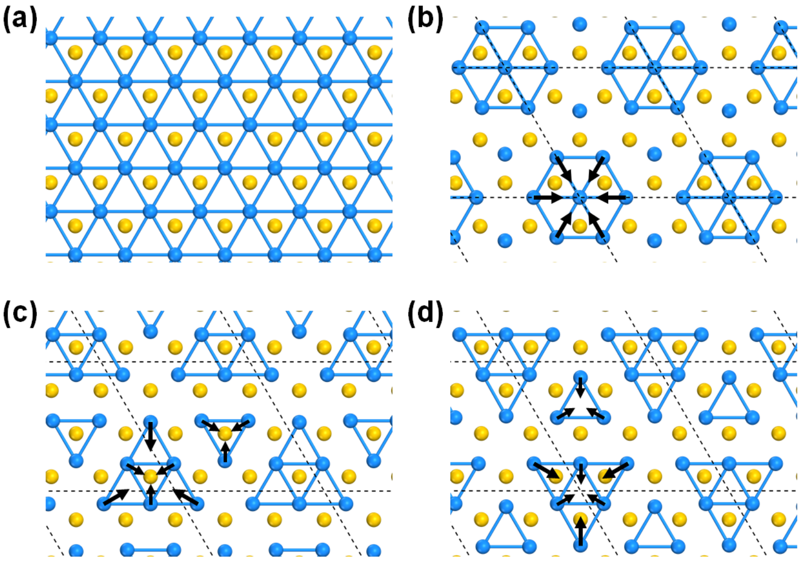

Supplementary Figure 1: **CDW phases on** 3×3 **monolayer 1H-TaS$_2$.** Top view of atomic structures of monolayer 1H-TaS_2. Ta and S atoms are depicted as blue and yellow spheres, respectively. (**a**) triangular lattice in a 1×1 unit cell without CDW modulation. (**b-d**) selected CDW phases (#1 to #3 in ??, respectively) on 3×3 monolayer 1H-TaS_2. Atomic displacements and unit cells are illustrated as black arrows and black dashed lines, respectively. Configuration (**d**) has the lowest formation energy of the selected configurations.

In principle, both layers of the considered 1T/1H-TaS_2 heterostructure are subject to CDW modulations. In particular, the 1T layer features a $\sqrt{13} \times \sqrt{13}$ CDW and the 1H layer a 3×3 supercell. We now show that the latter has only marginal effect on the physics of the system and can therefore be safely neglcted. To quantify the effect of the 1H layer 3×3 CDW on the bilayer 1T/1H-TaS_2 system, we investigate the work functions of 1T- and 1H-TaS_2 monolayers with and without CDW modulation, and calculate the charge transfer in the bilayer system. The selected 3×3 CDW phases on 1H [1–3] as illustrated in Supplementary Fig. 1, with the relative work functions listed in Supplementary Table ??.

The results show that all considered 3×3 CDWs produce no notable variations in the work function, as it can be easily evinced from the fourth column of the table, listing the formation energies per formula unit ΔE/f.u, where the most stable configuration has value 0. In contrast, the formation of SoD on 1T results in a sizable 53 meV difference (readily obtainable comparing the values relative to the $\sqrt{13} \times \sqrt{13}$ and 1x1 1T structures). We also performed a Bader charge analysis to investigate the effect of CDW on the charge transfer between the two layers and find it to be comparable despite the formation of distinct CDW phases. This result indicates that the 3×3 CDW on the 1H layer can be neglected in the investigation of the electronic properties of the bilayer system and only the CDW in the 1T layer plays an essential role.

SUPPLEMENTARY NOTE 2. EFFECT OF A SUBSTRATE

In experimental realizations, it is common to make use of supporting platforms such as multilayer graphene [4, 5] or bulk 2H-TaS_2 crystal [6]. In the latter case, it is easy to notice that bulk 2H-TaS_2, used as a combined electron reservoir+substrate beneath monolayer 1T-TaS_2, has the same work function as monolayer 1H-TaS_2. It is more intriguing to investigate the effect of a graphene substrate on the flat bands in 1T/1H-TaS_2. For that reason, we employed in DFT simulation a 5×5 supercell of graphene, which commensurates with a $\sqrt{13} \times \sqrt{13}$ bilayer 1T/1H-

	unit cell	work function [eV]	ΔE/f.u. [meV]
monolayer 1H-TaS_2	1×1	5.905	3.28
	3×3 CDW #1	5.902	1.43
	3×3 CDW #2	5.907	0.66
	3×3 CDW #3	5.896	0
monolayer 1T-TaS_2	1×1	5.070	22.51
	$\sqrt{13} \times \sqrt{13}$ CDW	5.123	0
	unit cell	charge transfer [$e/(TaS_2)_{26}$]	ΔE/f.u. [meV]
bilayer 1T/1H-TaS_2	1×1	0.44	12.3
	3×3 (CDW on 1H)	0.43	11.4
	$\sqrt{13} \times \sqrt{13}$ (CDW on 1T)	0.41	0

Supplementary Table I: **Effect of CDW modulation on the charge transfer in bilayer 1T/1H-TaS_2.** Calculated work functions of monolayer 1H-TaS_2 and 1T-TaS_2 in absence and presence of typical CDW phases on 3×3 monolayer 1H-TaS_2. Formation energy differences per formula unit between identical polytypes are listed. $\Delta E = 0$ indicates the most stable configuration. The work function difference between the metallic 1×1 1T and the Mott-insulating $\sqrt{13} \times \sqrt{13}$ 1T structures is 53 meV, whereas the CDW on metallic H-TaS_2 does not produce significant differences. Also shown is the calculated charge transfer that occurs when 1T and 1H layers combine to form a bilayer structure ($(TaS_2)_{26}$) with CDW modulation in each layer.

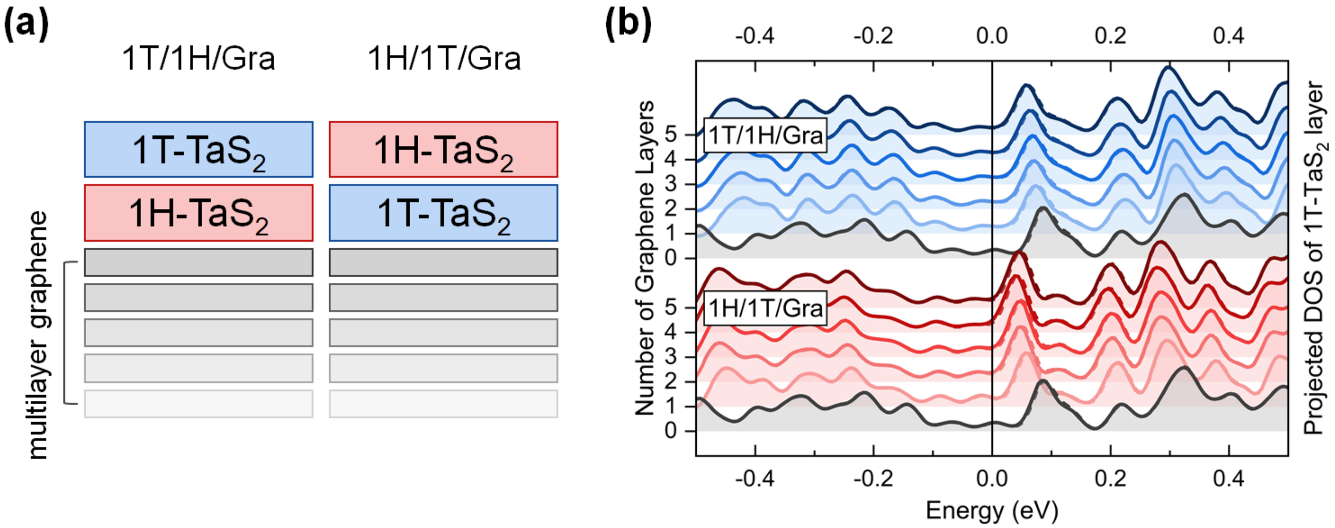

Supplementary Figure 2: **The effect of graphene substrate on the flat bands in 1T/1H-TaS_2.** (**a**) Schematic illustration of stacked layers of bilayer 1T/1H-TaS_2 on multilayer graphene used in the experiment [4] (**b**) DOS profiles of the 1T-TaS_2 layer in 1T/1H/Gra and 1H/1T/Gra systems with a different number of graphene layers. Zero (0) graphene layers indicate the free-standing bilayer 1T/1H-TaS_2. Optimized interlayer distances between 1T and 1H layers in the two systems are 5.78 Å and 5.81 Å, respectively, which have not changed considerably from the free-standing 1T/1H bilayer with 5.81 Å of separation. When the bilayer TaS_2 is placed on a graphene substrate that provides electrons to the bilayer, the degenerate empty flat bands shift slightly toward the Fermi level, but the electron doping provided by the multilayer graphene substrate is not sufficient to compensate the strong change transfer from the 1T to the 1H layer.

TaS_2 within 2% of lattice constant mismatch, where we used the lattice constant of free-standing bilayer 1T/1H-TaS_2 when constructing the 1T-1H-multilayer graphene (or 1H-1T-graphene) stacked layer systems. Fig. 2 depicts the DOS profiles of the 1T-TaS_2 layer in the complex structure. As the graphene layers serve as an electron dopant for the bilayer, placing the bilayer on a graphene substrate shifts the degenerate empty flat bands towards the Fermi level. Although a thicker multilayer graphene substrate provides more electrons to the bilayer system, the degenerate empty flat bands do not shift further, and we didn't observe any peak splitting coming from electron doping. However, it should be noted that the peak shift differs significantly between two stacking orders. When the 1T layer is positioned

between the 1H layer as an electron reservoir and the graphene substrate as an electron dopant, a larger peak shift is observed than when the 1T layer is placed above the 1H layer and far from the graphene layers. This result indicates that the flat band splitting in 1T-TaS_2 can be controlled not only by the interlayer distance, but also by considering adequate substrate platforms if sufficient electrons are supplied to cause the splitting (i.e. to dope the flat band).

SUPPLEMENTARY NOTE 3. INTERLAYER DISTANCE EFFECT ON CHARGE TRANSFER

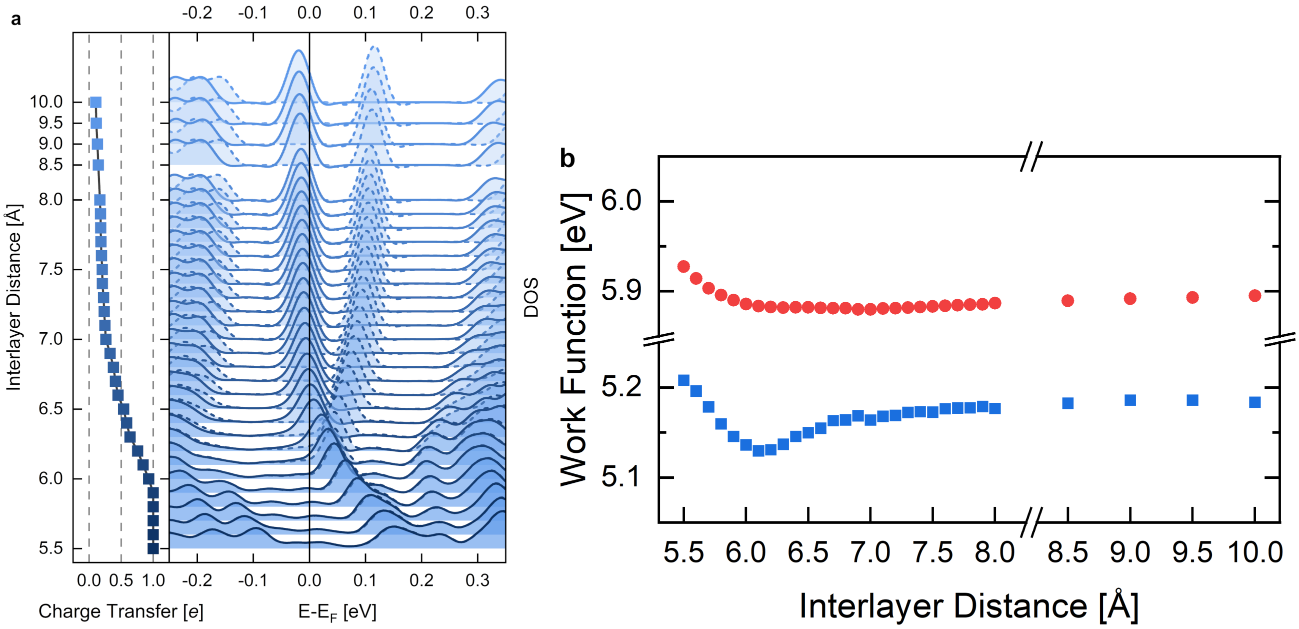

Supplementary Figure 3: **(a)** Interlayer distance-dependent DOS and charge transfer in the 1T/1H-TaS_2 bilayer up to 10.0 Å. **(b)** 1T- and 1H-side work function of bilayer 1T/1H-TaS_2 varied by interlayer distance.

As argued in the main text, interlayer separation, which is an intrinsic feature of each experimental realization, is of paramount importance on the entity of charge transfer. Here we detail the relation between the two, as summarized in Fig. 3. On the left, we show the behavior of the DOS and charge transfer as a function of interlayer distance in a larger range than what is plotted in Fig.2 of the main text. It is immediate to see how the charge transfer approaches 0 for interlayer distances exceeding 7Å. In this regime, the system effectively behaves as a plane-plate capacitor, and the two layers are uncoupled.

In the right panel of Fig. 3 we show the work function, a difference between Fermi level and vacuum electrostatic potential, calculated in the framework of DFT. Due to the fact that two layers are placed in one unit cell, in DFT calculations they share a globally defined Fermi level but show different vacuum electrostatic potentials at both surface. The 1T-side of the bilayer heterostructure shows a steeper decrease of work function than the 1H-side when d_{int} <6.1 Å. The work function of the 1T-side increases as the flat-band filling factor increases, whereas the 1H-side exhibits a rather converged work function. At d_{int} >7.5 Å, the two layers both show converged values. In general, the interlayer distance dependence of the work function is rather weak across the range.

SUPPLEMENTARY NOTE 4. ATOMIC RELAXATION

For our DFT and DMFT calculations, we employed the optimized structures with entirely relaxed interatomic bondings and lattice constants, fixing the interlayer distance and assuming as the only constraint the positioning of Ta atoms on each 1T and 1H layer in two parallel planes. For example, in the case of d_{int}=5.8 Å, Ta atoms on 1T and 1H layers are fixed at z=0.0 Å and z=5.8 Å, respectively. However, the x and y components of Ta atoms were freely relaxed to permit CDW modulation, and the x, y, and z components of all S atoms were also freely optimized. The result is shown in Fig. 4(c). We have also investigated the atomic displacement in CDW modulation upon varying the interlayer distance. Because the 3×3 charge order in the 1H-TaS_2 has a negligible impact on the charge transfer

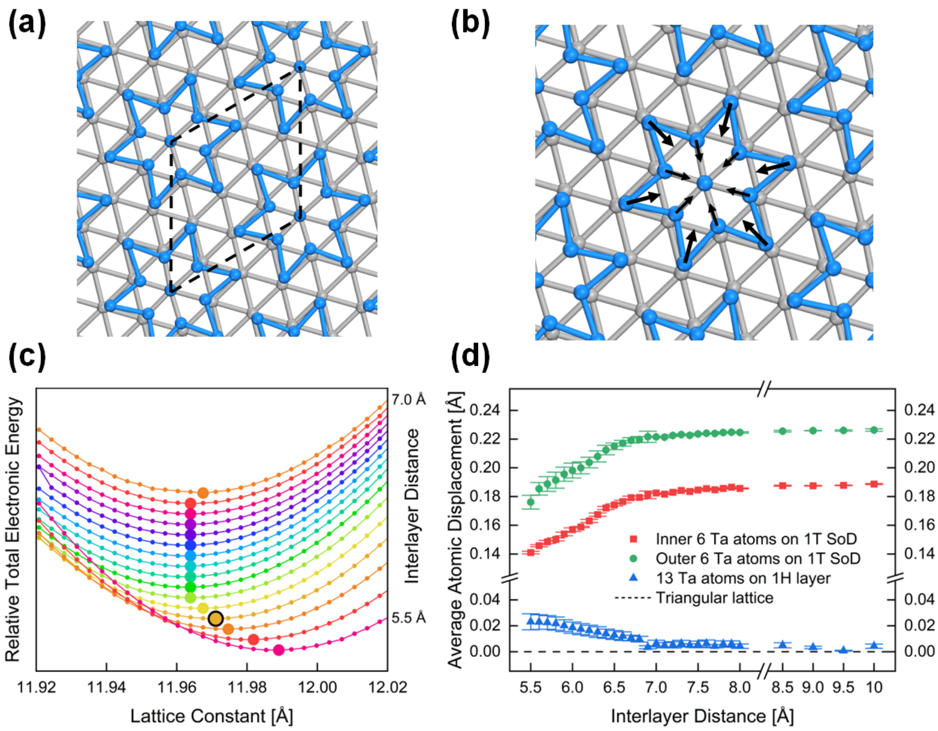

Supplementary Figure 4: **Formation of the Star-of-David on the 1T layer in bilayer 1T/1H-TaS$_2$ with varying interlayer separation.** (**a**) The unit cell of $\sqrt{13}\times\sqrt{13}$ bilayer 1T/1H-TaS$_2$ structure is shown by black dashed lines. Ta atoms on 1T and 1H layers are illustrated in blue and gray, respectively. (**b**) Atomic displacements of Ta on 1T layer in CDW modulation, whose direction is represented by black arrows. (**c**) Energy-lattice constant relation in $\sqrt{13}\times\sqrt{13}$ bilayer 1T/1H-TaS$_2$ at fixed interlayer separations. Each energy curves are vertically offset for clarity and shown in arbitrary units. Energy minima is depicted as large filled circles, and the global energy minima at 5.8 Å of interlayer distance, of our all explored geometries, is marked with a black edge. (**d**) Atomic displacements of Ta on the 1T and 1H layers as a function of interlayer distance. The average deviations from a perfect triangular lattice are shown with 95% level of confidence. As interlayer spacing widens and the hybridization decreases, the 1H layer reverts to a triangular lattice, whereas the 1T layer shrinks significantly more.

between layers (see previous sections), we have focused on the formation of SoD on the 1T layer using a $\sqrt{13}\times\sqrt{13}$ supercell. Nevertheless, we have optimized 1H layers to obtain relaxed structures under the given conditions. For greater interlayer distances d_{int} >7.0 Å, the lattice constant was fixed at 11.964 Å of converged value. As depicted in Fig. 4(d), Ta atoms on the 1T layer form SoD even at a short interlayer distance of d_{int}=5.5 Å by being slightly shifted from the triangular lattice. The degenerate empty localized states, caused by this minor shift at d_{int}=5.5 Å are observed above the Fermi level. As the interlayer distance increases, the hybridization decreases and the size of the SoD reduces as the displacement increases. The SoD size has converged at $d_{int}\approx 7.5$ Å.

SUPPLEMENTARY NOTE 5. CONSIDERATIONS ON THE LONG-RANGE INTERACTION TERMS

The effect of non-local (e.g. intra-layer, inter-later nearest neighbor) interaction terms cannot be dynamically captured by single-site DMFT. We argue, however, that their effect is not large enough to make our approximation inaccurate. We can estimate the entity of these corrections at the mean-field level by comparing various correlation functionals, as done in Fig. 5, where we employ PBE+U, r^{2}SCAN+rVV10 [7], and local-mBJ [8]. Only the value U_{eff} = 2.27 eV, calculated using the linear response method for Ta-5d orbitals [9], was used in the PBE+U calculation. r^{2}SCAN+rVV10 and local-mBJ functionals were selected due to their strength in the implementation of vdW interaction and non-local exchange. Although there are slight variations in the magnitude of the peak splitting, the interlayer spacing where the splitting begins, and the shape of flat band peaks, we observe that the overall trends are not significantly changed in comparison to pure PBE results.

While we cannot calculate the intercluster Coulomb repulsion from first principles, we can roughly estimate the

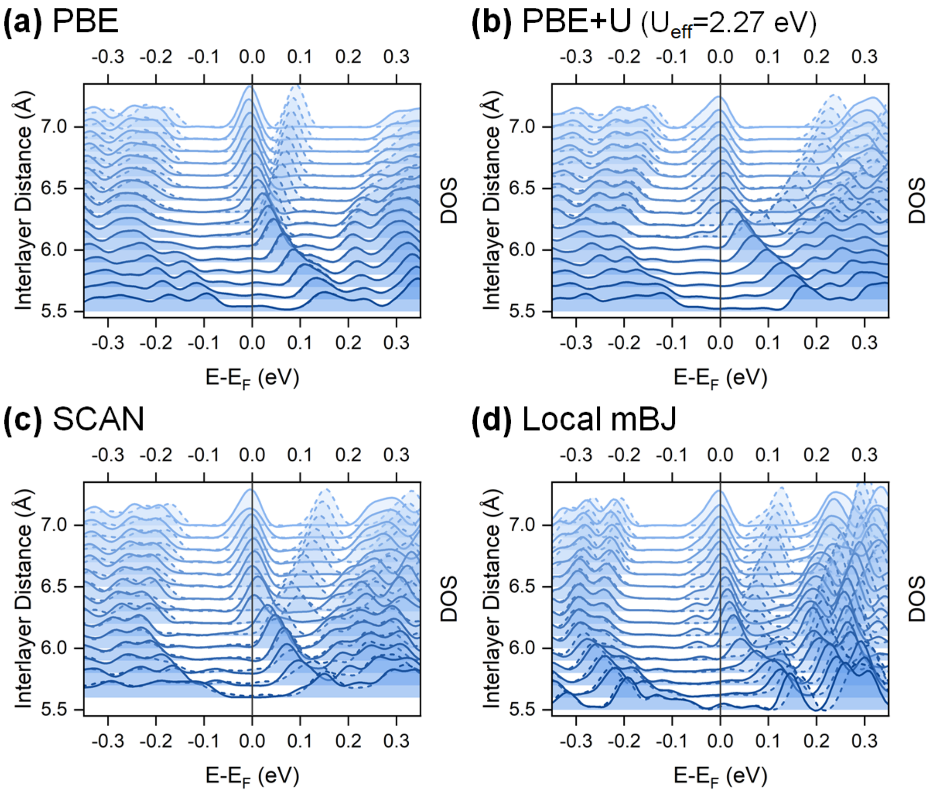

Supplementary Figure 5: **Interlayer spacing-dependent evolution of the Projected Density of States of the 1T-TaS$_2$ layer at the GGA and meta-GGA level of theories.** DOS of the 1T-TaS$_2$ layer in the bilayer 1T/1H-TaS$_2$ structure for a range of d_{int} computed by using (**a**) pure PBE (from the manuscript), (**b**) PBE+U (U_{eff}=2.27 eV), (**c**) r^{2}SCAN+rVV10, and (**d**) local-mBJ functionals. Contributions corresponding to majority and minority spins are illustrated as solid and dashed lines, respectively. Corrections for self-interaction error and a drawback resulting from the adoption of *averaged* exchange-correlation energy manifest in some distinct splitting strengths. However, the interplay between the 1H layer as a spin reservoir and the 1T layer as a localized spin carrier does not change.

effect of screening. First of all, the distance between the cluster centers is about $d \approx 1.22$ Å, which gives an unscreened V on the order of 1 eV. The fact that there is some spectral weight on the non-central Ta atoms modifies this number very little, as multipole interactions of the 6th order are small.

Let us first discuss the effect of screening from the 1H layer: the charges to be screened are *outside* the 1H layer, therefore a correct account of this process involves the use of image charges, and as a result, contrary to what is implicitly implied in the Kondo model, the screening is not exponential but of a dipole-dipole type. Furthermore, the length of the dipole is of the same order as d, so the reduction of V due to the 1H screening is rather small.

The intralayer 1T screening has also to be taken into account. We have calculated it in DFT+U for an undoped 1T layer. In this case the screening is semiconducting and $\epsilon_0 \approx 12$. This reduces V to less that 100 meV. Additional screening from mobile electrons in the lower Hubbard band, with is harder to estimate, is expected to entail an additional substantial reduction of V.

It is furthermore worth noting that, if one does include V on a mean field level, the result is a trivial shift of the chemical potential, and the remaining effects, which could, in principle, modify our calculations, only come from residual deviations from the mean field behavior, which are comparatively rather small.

For these reasons, we believe that our neglecting of the intersite Coulomb effect is justified, especially given that our main conclusions are of qualitative nature (quantitative details may depend on the quality of the interface).

SUPPLEMENTARY NOTE 6. FERMI SURFACE AND SPECTRAL FUNCTION

From a simple comparison of the susceptibilities in figure 3 of the main text, it is not immediate to evince the difference between the heavy Fermion and doped Mott scenarios: for example, the susceptibilities at $CT = 1/2$,

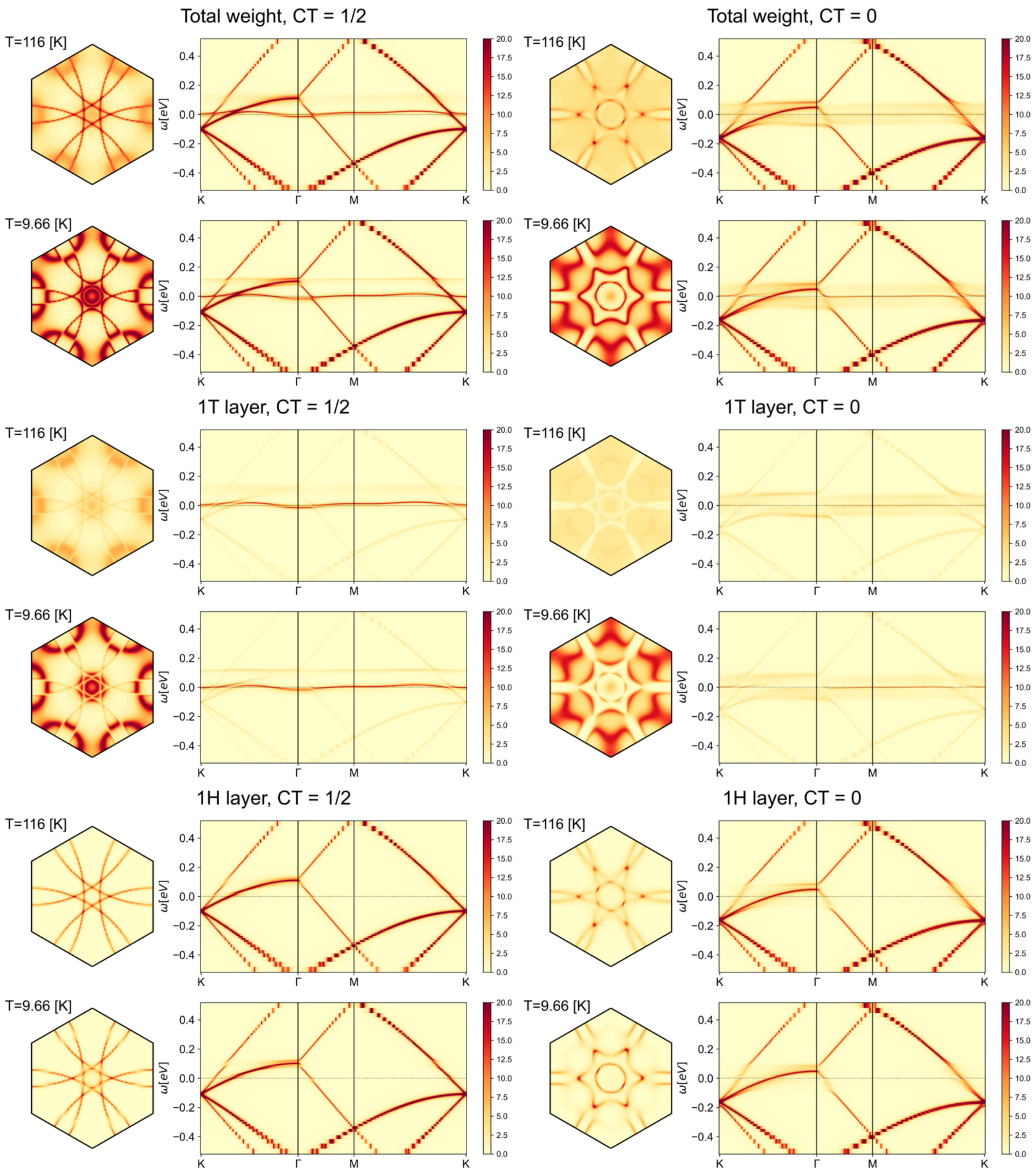

Supplementary Figure 6: **Comparison of total, 1T and 1H Fermi surface and momentum-resolved spectral funtion** $A(\mathbf{k}, \omega)$. Data for the two parameters sets $CT = 1/2$, $V1 = 30$ meV and $CT = 0$, $V1 = 100$ meV at temperatures $T = 116$ K and 9.66 K.

$V1 = 30meV$ and $CT = 0$, $V1 = 100meV$ seem to be qualitatively analogous. This similarity is however deceptive: indeed, a big difference between the two scenarios can be evidenced already by comparing the instantaneous local moment, shown in the inset of Fig. 3 for $\tau = 0$: in fact, in the presence of charge transfer ($CT = 1/2$) its value is already dramatically screened (less than 0.5, inset Fig. 3 a), while it is considerably less so in the $CT = 0$ (no charge transfer) case (inset Fig. 3 b). To corroborate the statement on the fundamental difference between the doped Mott ($CT = 1/2$, $V1 = 30$ meV) and heavy Fermion ($CT = 0$, $V1 = 100$ meV) scenarios, we compare the momentum-resolved spectral functions ($A(\mathbf{k}, \omega)$), which are experimentally measurable via angular resolved photoemission spectroscopy (ARPES). We show total and layer resolved spectral functions at temperatures $T = 116$ K and 9.66 K in Fig. 6. A clear distinction between both scenarios can be seen from the spectral functions along a high-symmetry path and most clearly from the spectral weight maps at the Fermi energy ($A(\mathbf{k}, \omega = 0)$).

In the ($CT = 0$, $V1 = 100$ meV)-case we find at the higher temperature incoherent spectral weight at the Fermi level originating from the T-layer and from the H-layer bands at the Fermi level. When coherence in the T-layer develops at low temperature the apparent Fermi surface completely reconstructs with new Fermi surface segments originating from coherent quasiparticles appearing in the T-layer. The strong hybridization between T and H-layer states can be inferred from avoided crossings of the low-energy quasi particle bands and from the character change (T-layer vs H-layer) along the Fermi surface segments appearing at low temperature. Such a temperature induced reconstruction of the Fermi segments is indeed characteristic of heavy Fermion systems.

The ($CT = 1/2$, $V1 = 30$ meV) case differs from this scenario. While some sharpening of spectral features upon lowering the temperature appears also here, we observe at the Fermi level T-layer-derived coherent spectral weight already at the higher temperature and there is no complete reconstruction as is the case for $CT = 0$, $V1 = 100meV$. This can be most clearly seen in the H-layer Fermi level spectral weight, which remains almost unchanged with temperature ($CT = 1/2$, $V1 = 30$ meV)-case — in strong contrast to the ($CT = 0$, $V1 = 100$ meV)-case.

SUPPLEMENTARY NOTE 7. INTERLAYER ORBITAL HYBRIDIZATION

In developing the tight-binding Hamiltonian for the heterostructure model, we relied on the hypothesis that the out-of-plane d_{z^2} orbitals contribute the most to the inter-layer hybridization. We provide a visual confirmation of this fact by introducing an estimator for the hybridization of different orbitals defined as

$$\Omega(\phi_\alpha, \phi_\beta) \coloneqq |\langle \phi_\alpha | \Psi_{nk} \rangle| \cdot |\langle \phi_\beta | \Psi_{nk} \rangle|$$

where $\phi_{\alpha,\beta}$ are the chosen d orbitals and Ψ_{nk} is the Kohn-Sham wavefunction from the DFT calculation near the Fermi level. The results are shown in Fig. 7, where the orbital characters corresponding to the largest values of Ω are plotted. As expected, the overlap between the d_{z^2} orbitals in the 1H and 1T layers is noticeably larger than the others, including the second largest ($d_{x^2-y^2}(H)$,$d_{z^2}(T)$).

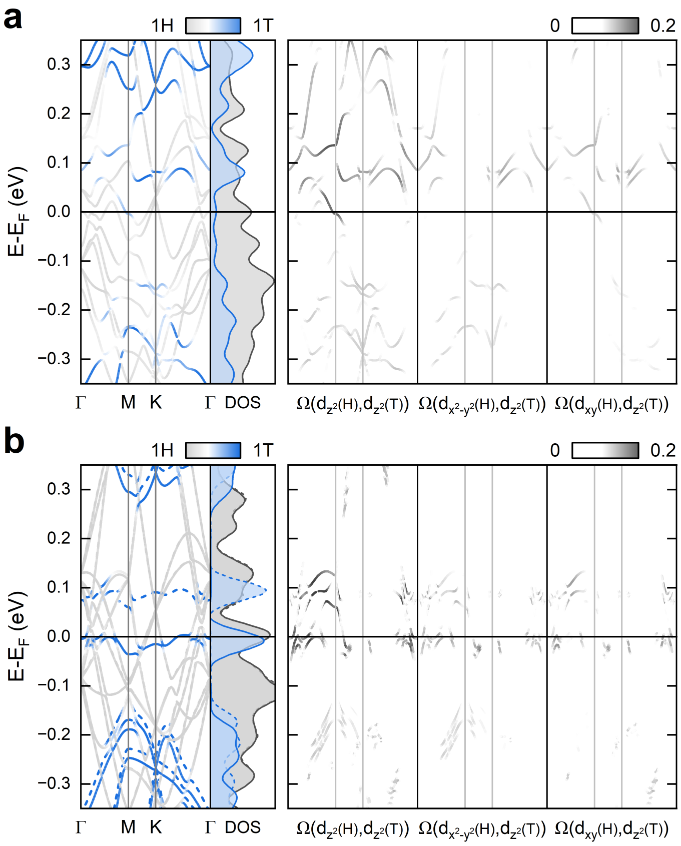

Supplementary Figure 7: Hybridization estimator between different orbitals. Orbital hybridization estimator for an inter-layer distance of (**a**) 5.8 Å and (**b**) 7.0 Å. The panels on the left replicate Fig. 2a,b in the manuscript at the two chosen interlayer distances. On the right we show the DFT band structure weighted with the hybridization estimator Ω for the largest contributions to the overall hybridization.

[1] J. A. Wilson, F. J. Di Salvo, and S. Mahajan, Charge-density waves in metallic, layered, transition-metal dichalcogenides, Phys. Rev. Lett. **32**, 882 (1974).

[2] J. A. Wilson, More concerning cdw phasing in 2h-$TaSe_2$, Journal of Physics F: Metal Physics **15**, 591 (1985).

[3] Z. Wang, Y.-Y. Sun, I. Abdelwahab, L. Cao, W. Yu, H. Ju, J. Zhu, W. Fu, L. Chu, H. Xu, and K. P. Loh, Surface-limited superconducting phase transition on $1T-TaS_2$, ACS Nano **12**, 12619 (2018).

[4] V. Vaňo, M. Amini, S. C. Ganguli, G. Chen, J. L. Lado, S. Kezilebieke, and P. Liljeroth, Artificial heavy fermions in a van der waals heterostructure, Nature **599**, 582 (2021).

[5] W. Wan, R. Harsh, A. Meninno, P. Dreher, S. Sajan, H. Guo, I. Errea, F. de Juan, and M. M. Ugeda, Evidence for ground state coherence in a two-dimensional kondo lattice, Nature Communications **14**, 7005 (2023).

[6] C. G. Ayani, M. Pisarra, I. M. Ibarburu, M. Garnica, R. Miranda, F. Calleja, F. Martín, and A. L. Vázquez de Parga, Probing the phase transition to a coherent 2d kondo lattice, https://onlinelibrary.wiley.com/doi/pdf/10.1002/smll.202303275.

[7] J. Ning, M. Kothakonda, J. W. Furness, A. D. Kaplan, S. Ehlert, J. G. Brandenburg, J. P. Perdew, and J. Sun, Workhorse minimally empirical dispersion-corrected density functional with tests for weakly bound systems: $r^2SCAN + rVV10$, Phys. Rev. B **106**, 075422 (2022).

[8] T. Rauch, M. A. L. Marques, and S. Botti, Local modified becke-johnson exchange-correlation potential for interfaces, surfaces, and two-dimensional materials, Journal of Chemical Theory and Computation **16**, 2654 (2020).

[9] P. Darancet, A. J. Millis, and C. A. Marianetti, Three-dimensional metallic and two-dimensional insulating behavior in octahedral tantalum dichalcogenides, Physical Review B **90**, 045134 (2014).
